# A Festschrift for Stephen L. Wood

**DOI:** 10.3897/zookeys.56.513

**Published:** 2010-09-17

**Authors:** Anthony I. Cognato, Miloš Knížek

**Affiliations:** 1 Michigan State University, USA; 2 Forestry and Game Management Research Institute, Czech Republic

We intended this Festschrift as a celebration of Dr. Stephen L. Wood’s 85^th^ birthday. Sadly Dr. Wood past away a little before his 85^th^ birthday thus this volume pays tribute to his life’s contribution to the study of bark and ambrosia beetles, which is summarized in the next article by Donald Bright. We arranged the volume geographically, starting with taxonomy of North American scolytines, followed by articles on new species introductions, biology, phylogeny. The articles treat species throughout the world, which reflects Dr. Wood’s breadth of taxonomic knowledge. The contributing authors also reflect worldwide taxonomic expertise. Dr. Wood personally influenced and trained three generations of scolytine taxonomists, including some of these authors. He left a lasting impression on the careers of several of us, which we relay below.

**Figure 1.**
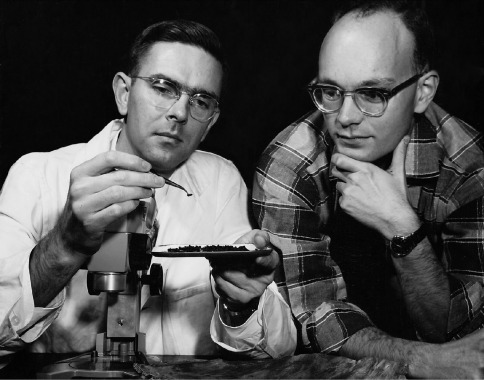
Steve Wood and his first graduate student, Don Bright, checking some beetles, 1960.

Donald E. Bright: I first met Steve in 1957 while I was serving in the U. S. Army. Previously, I had been attending Colorado A & M College (now Colorado State University) where I met T. O. (Ted) Thatcher, a professor in the Department of Entomology. Ted became a friend in addition to an advisor and mentor. He mentioned that his brother-in-law was interested in bark beetles and had just accepted a position at BYU. During my Army service, I was fortunate to be stationed at Dugway Proving Grounds, Utah and I took the first opportunity I had to visit Steve at BYU. During the next two years, I, often with Steve, collected throughout Utah. I also was able to occasionally take my specimens to BYU to have Steve check my identifications. I especially remember one trip to the La Sal Mountains in southern Utah to collect a bark beetle species, Pityoborus secundus Blackman, known at that time from a few specimens from that one locality. On the 4^th^ of July, 1958, Steve and I drove to the La Sal Mountains in my old car. We found the beetles in some shaded-out branches of a Ponderosa pine at the bottom of a steep canyon. On the way out, the fuel pump on my car gave out and we barely made it out of the canyon. We spent that night somewhere near Moab, Utah, with me under the car replacing a fuel pump and Steve sleeping in the motel. Many other collecting trips were made which influenced and enhanced my enthusiasm for the study of this group of beetles. As a result of these experiences, I decided to attend BYU for my Masters Degree with Steve as my major advisor (Fig. 1).

**Figure 2.**
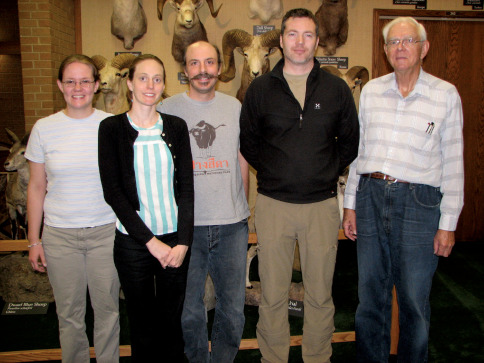
Three generations of scolytine systematists visiting Dr. Wood at BYU. From left to right: Sarah Smith, Stephanie Dole, Anthony Cognato, Bjarte Jordal and Stephen Wood, 2007.

Anthony I. Cognato: I first corresponded with Dr. Wood as a Ph.D. student in 1996. He offered thoughts and advice on Ipini taxonomy and leant me a rare book. Our correspondences were infrequent but substantial throughout my career. Most memorable was an allegory pertaining to molecular systematics where he described Colonel Thomas Casey’s use of advanced optics of the late 1800’s to see new morphological variation and to describe hundreds of beetle species and genera, which are now synonymized. I thanked him for the advice and assured him that my education in molecular systematics included the interpretation of DNA data for taxonomic decisions. Thus, we didn’t always agree on taxonomic matters but Pseudips was our only major disagreement and our personal meetings were always friendly and professional. He was particularly helpful with the development of my career through the donation of specimens and participating in my NSF-PEET study of tropical xyleborines through the mentorship of my students (Fig. 2). His guidance will be missed.

**Figure 3.**
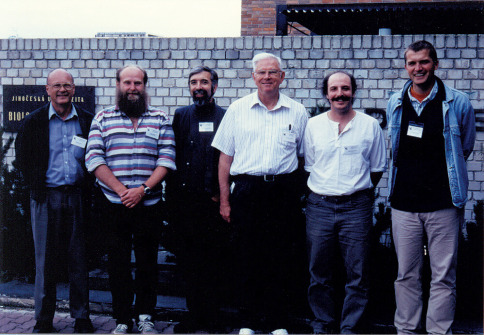
A few scolytine systematists at the VIth European Congress of Entomology, České Budějovice, Czech Republic, August 23–29, 1998. From left to right: Donald E. Bright, Miloš Knížek, Lawrence R. Kirkendall, Stephen L. Wood, Anthony I. Cognato, Christian Stauffer.

**Figure 4.**
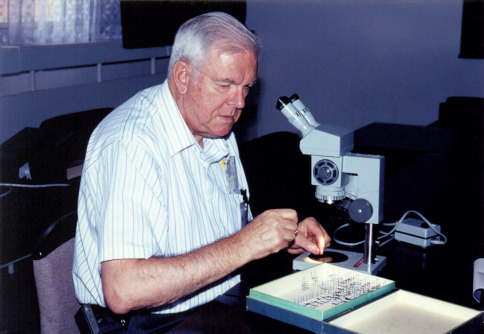
Stephen L. Wood at the “Bark and Timber Beetles Workshop” at the VIth European Congress of Entomology, Ceské Budejovice, Czech Republic, August 23–29, 1998.

Miloš Knížek: My first contact with Professor Stephen L. Wood was dated rather long ago, in the beginning of 1980’s, ten years after the start of my interest in bark beetles taxonomy. My dream, to meet him personally, was not possible in that time, because of the political situation in Czech Republic. With help of my father I contacted Steve by a letter, asking for some reprints. Steve immediately and fully supported me with his published studies and this support continued throughout my career. Early after the „velvet revolution“ in our country, when our borders were finally open to the world, I made an effort to meet as many specialists in bark beetle taxonomy as possible. On the occasion of the VIth European Congress of Entomology, which was organized by my colleagues in the Entomological Institute in Ceské Budejovice (Czech Republic) in 1998, I decided to organize the workshop on bark and timber beetles taxonomy to bring all these specialists together and meet them at the same time (Fig. 3). With help of Professor Antonín Pfeffer, the world wide known bark beetle taxonomist of palaearctic species and my "private teacher" in this field, we put together the list of possible participants this historical meeting. Unfortunately, Professor Pfeffer passed away suddenly, at 93. I continued to prepare the meeting with help of Larry Kirkendall and invited 55 specialists from around the world. This was my first occasion to personally meet Steve Wood. Despite his age Professor Wood impressed me with his enthusiasm and perfect knowledge of bark beetles (Fig. 4). We developed a close professional relationship and he soon invited me to visit his collection in Provo (Monte L. Bean Life Science Museum, Brigham Young University). Within one year with much help from Don Bright I went to BYU. I spent my whole visit working closely with Professor Wood where we engaged in motivating discussions of scolytine taxonomy. I stayed with Steve and his wife and got to know them personally. I spent many unforgettable moments with both of them. Steve's wife was very caring and very apprehensive that I would get hurt, during my weekend bark beetles collecting trips. She always told me: "Milos, please, do not climb the rocks in the mountains, many people died by falling off them!". Perhaps my answers and promises were not satisfactory enough, so, Steve added: "I am sure he will not climb the rocks, there are no bark beetles in the rocks". Meeting Professor Wood had exceptional influence on my scientific study as well as on my whole life. It is my honour to be the co-editor of this festschrift even though it is only a small repayment for what I learned from Steve.

Sarah M. Smith: I had the privilege of meeting Dr. Wood nine months into my graduate studies with Dr. Cognato. Although my visit at Brigham Young University lasted only a few days, Dr. Wood made a lasting impression on me. Throughout the rest of my master’s degree, I remained in close contact with Dr. Wood and we had phone calls about every three months, the last of which was less than a month before he passed. His advice, both in person and on the phone, was profound and he always encouraged me in my studies. It is truly unfortunate that he passed away before the completion of my revision of Camptocerus, of which he very much wanted to see. I feel honored to work on a group of organisms that have had so much work done by a single individual. Dr. Wood continues to guide me in my work through his publications and I am motivated by his example. His contribution to the taxonomy of bark and ambrosia beetles was truly a remarkable feat.

Dr. Wood was delighted with the knowledge that we were preparing a Festschrift in his honor. We like to think that he would have been happy with this publication.

